# Engineering Cell Surfaces with Polyelectrolyte Materials for Translational Applications

**DOI:** 10.3390/polym9020040

**Published:** 2017-01-28

**Authors:** Peipei Zhang, Michelle L. Bookstaver, Christopher M. Jewell

**Affiliations:** 1 Fischell Department of Bioengineering, University of Maryland, College Park, MA 20742, USA; pzhang14@umd.edu (P.Z.); mlbooks@umd.edu (M.L.B.); 2 Department of Microbiology and Immunology, University of Maryland School of Medicine, Baltimore, MA 21201, USA; 3 Marlene and Stewart Greenebaum Cancer Center, Baltimore, MA 21201, USA; 4 United States Department of Veterans Affairs, Baltimore, MA 21201, USA

**Keywords:** polyelectrolyte, multilayer, cell modification, sensing and signaling, drug delivery, vaccine and immunotherapy, surface protein, tissue engineering, regenerative medicine

## Abstract

Engineering cell surfaces with natural or synthetic materials is a unique and powerful strategy for biomedical applications. Cells exhibit more sophisticated migration, control, and functional capabilities compared to nanoparticles, scaffolds, viruses, and other engineered materials or agents commonly used in the biomedical field. Over the past decade, modification of cell surfaces with natural or synthetic materials has been studied to exploit this complexity for both fundamental and translational goals. In this review we present the existing biomedical technologies for engineering cell surfaces with one important class of materials, polyelectrolytes. We begin by introducing the challenges facing the cell surface engineering field. We then discuss the features of polyelectrolytes and how these properties can be harnessed to solve challenges in cell therapy, tissue engineering, cell-based drug delivery, sensing and tracking, and immune modulation. Throughout the review, we highlight opportunities to drive the field forward by bridging new knowledge of polyelectrolytes with existing translational challenges.

## 1. Introduction

The term “cell surface engineering” refers to the modification of cell surfaces with synthetic or natural materials to modulate cell function. The purpose of this review is to describe how polyelectrolyte materials can be used in cell surface engineering to address some of the clinical challenges in translational ideas from the benchtop to the clinic. We begin with a brief background on cell surface engineering, along with highlights of the current clinical opportunities. Next we introduce polyelectrolytes and present current and future opportunities for this class of materials to be exploited in overcoming challenges in the biomedical field by pushing the forefront of cell surface engineering.

### 1.1. Introduction to Cell Surface Engineering

Many biological surfaces are composed of combinations of lipids, proteins, and carbohydrates that serve as the primary means by which cells transmit environmental cues for signaling. These cascades signal to and between cells to perform specific functions. The rapid development of new biological tools has helped to identify a plethora of pathways that can be harnessed to direct such processes. Cell receptors and their respective ligands, downstream signaling pathways, and external stimuli can be exploited to regulate cellular functions spanning survival, proliferation and differentiation, and metabolism. Cell surface engineering utilizes engineering, chemistry, and biology techniques to conjugate cell surface molecules with natural ligands, functional biological components, or synthetic materials.

Surface-engineering is already being applied to many clinical areas using approaches that include polyelectrolytes and many other strategies. Conjugating exogenous ligands (i.e., those that don’t naturally occur in a cell or tissue) onto cell surfaces can alter molecular pathways within cells, causing changes in functions such as cell adhesion, migration, and proliferation [1,2,3]. For transplantation animals or patients, conjugating biological molecules onto cell surfaces can mimic a supportive microenvironment, which can be harnessed to improve the longevity and therapeutic outcomes of cells once injected [4,5]. Additionally, surface modification with immune inhibitory molecules allows implanted cells to avoid clearance by the immune system [6,7]. New technical developments have also allowed cell surface engineering to expand to more fields. For example, in tissue engineering, cell surface engineering is being used to develop novel tissue architectures composed of specific cell types at different spatial layers; this allows recapitulation of more realistic tissue structures [8,9,10,11]. In drug delivery, cells loaded with therapeutic cargos can pass through biological barriers such as the endothelium or stromal tissues that provide supportive functions [12]. This is very different from conventional drug delivery methods, where most delivery vehicles rely on passive mechanisms such as non-specific accumulation through leaky blood vessels walls to target tumors (i.e., enhanced permeation and retention (EPR)) [13]. In stem cell engineering, mesenchymal stem cells—cells able to differentiate into many specialized cell types—are chemically modified with cell homing receptors on their surfaces to improve targeting to specific tissues. These studies address a significant barrier in the clinical application of stem cell therapies: the need for an adequate number of viable cells to reach the target tissue within short periods of time [14]. In early stage type I diabetes trials, for example, polyelectrolyte-modified islet cells have been studied as a route to restore healthy production of insulin—a molecule dysregulated during diabetes [15]. In cancer immunotherapy, a powerful new cell surface engineering technology is chimeric antigen receptor T cells (CAR-T). This vanguard idea allows modification of the surface receptors killer T cells to recognize cells being targeted for destruction, and has shown great potential in cancers such as acute lymphoblastic leukemia, multiple myeloma, and glioblastoma [16]. Further, the Food and Drug Administration (FDA) recently approved autologous (i.e., from the same individual) delivery of dendritic cells to treat cancer after being isolated and activated in cell culture outside of the patient. Similarly, autologous T cells expanded outside of patients and then reinfused are in clinical trials for the treatment of HIV, chronic infections, and other cancers [17].

### 1.2. Key Challenges in Cell Surface Engineering

Despite the potential of cell surface engineering, enormous challenges remain in designing effective and clinically-translatable technologies and therapies. Surface engineering should not impact the viability of the cells, and surface grafting or conjugation procedures must be performed without hindering normal cell functions or interactions between surface proteins or ligands. Surface modifications should also minimize altering cell membrane properties, such as the viscosity and elasticity of lipid bilayers, unless this is the goal. In addition, the technologies need to avoid exposure of cells to harsh conditions such as heat, extreme pH, shear force, and organic solvents. Unfortunately, these challenges are exacerbated by the diverse conditions that cells function in, the varied membrane compositions, and the contrast between the synthesis conditions of synthetic materials and cells. Thus, one of the major challenges in cell surface engineering is modifying specific cell surface molecules without compromising the normal functions or properties of other molecules, and the cells more generally.

As already alluded to, cells have a sophisticated dynamic structure, creating additional challenges for cell surface engineering [18]. For example, surface lipids and protein molecules continuously redistribute on the membrane and are involved in the processes of internalization or degradation of analytes, particles, debris, and other components cells encounter. This creates a new challenge because small molecules or materials on the cell surfaces may be internalized by the cells through uptake processes such as phagocytosis or endocytosis, thus abolishing or changing the function of the modification [19,20]. Further, material chemistry need to be compatible with in vitro (i.e., in cell culture) and in vivo (i.e., in animals or patients) applications of the engineered cells. Upon implantation, cells are exposed to shear stress and hydrodynamic forces during circulation in the blood, which can potentially damage the cells [21,22]. In addition, cells modified with exogenous materials may be rejected or attacked by a recipient’s immune system. Surface engineering processes should also avoid potential unregulated aggregation of engineered cells in vivo, which may induce blood platelets or clotting [23].

Genetic engineering is broadly studied as a technology for modulating cell functions and involves introducing or knocking out specific genes or target receptors [24,25]. Some of these goals involved engineering cells to express target molecules on surfaces. However, the clinical impact of genetic engineering has been largely unrealized owning to ineffective gene transfer, tedious and expensive transmission procedures, and safety concerns (e.g., genetic modification could drive oncogenes during the introduction of external genes to the target cells) [26]. Furthermore, despite the enormous investments by the drug delivery community, efficient gene delivery vehicles for use in humans remain elusive, and current clinical gene carriers rely on viruses (e.g., during vaccination) that carry additional potential safety issues. Another limitation is the cell surface cannot be directly modified with specific synthetic materials. Together, these challenges in genetic engineering have also helped motivate interest in alternative strategies to more rationally control the surface functions and molecules displayed by cells.

Many classes of materials have been explored for engineering cell surfaces, ranging from biological molecules such as proteins, to synthetic materials including scaffolds, magnetic or drug-carrying nanoparticles, and polyvalent molecules or polymers [27]. These materials are attached to the cell surfaces via antibody-specific interactions, hydrophobic interactions, hydrogen bonding forces, and electrostatic interactions. The versatility of polyelectrolytes, in particular, creates unique opportunities to engineer cell surfaces for translational goals [28].

### 1.3. Introduction of Polyelectrolyte Materials

**Figure 1:**
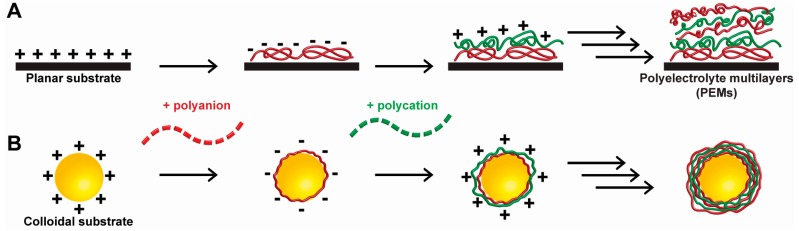
Schematic showing the process of layer-by-layer assembly (LbL) on a (**A**) planar or (**B**) colloidal substrate. Polycation and polyanion layers are deposited onto the substrates in alternating layers. The substrate is washed after the deposition of each layer to remove excessive materials from the substrates. These procedures are repeated until the desired number of layers are assembled. Adapted with permission from [36].

Polyelectrolytes are materials that contain a high level of ionizable groups along a polymeric backbone. These materials can be anionic, cationic or amphiphilic in charge, and include both synthetic and naturally occurring cargos. Due to their unique properties, polyelectrolyte materials have been widely used in the biomedical field, but this review is focused on their use in cell surface engineering [29,30]. Layer-by-layer (LbL) assembly is one of the most ubiquitous techniques for producing ultra-thin polyelectrolyte multilayer (PEM) films. PEMs were first developed by Decher et al. in 1997 [31,32,33,34,35]. This technique utilizes alternate deposition of oppositely charged materials onto a substrate to form ultra-thin PEMs by electrostatic interactions, shown schematically in Figure 1 [36]. Historically, electrostatic interaction was the major force used in LbL, but LbL assembly has evolved to include covalent bonding, hydrogen bonding, hydrophobic interactions, and coordination bonding [37]. The LbL technique allows for precise control over physical PEM properties, such as roughness, thickness, and porosity, as well as electrical, magnetic, mechanical, and optical properties [38]. These properties can be tuned by adjusting parameters during assembly including pH, ionic strength, and the concentration or composition of materials employed to form the PEMs. PEMs can be deposited on substrates of different length scales using planar substrates (e.g., glass, silicon), colloidal particles, or macroscopic devices (Figure 1) [36]. In addition to water-soluble polyelectrolyte materials, other components can also be incorporated into PEMs, ranging from nanoparticles—such as silica colloids, gold nanoparticles, metal oxide particles, to biological materials including viruses, proteins, nucleic acids, and immune signals (e.g., cytokines) [36,39,40]. LbL can be performed in environmentally-amenable solvents and offer the advantages of low-cost and biocompatible manufacturing [34]. Some polyelectrolyte materials can also form three-dimensional (3D) hydrogel networks upon specific chemical interactions [41]. Compared to other bulk materials, polyelectrolyte-based hydrogels have improved permeability of gases and nutrients. These materials can be utilized to engineer cells by encapsulating them in an artificial environment that allows the study of interactions between materials and cell surface receptors [42,43]. Together, this versatility makes polyelectrolytes an attractive choice for translational purposes in the biomedical engineering field.

### 1.4. Cell Surface Engineering with Polyelectrolyte Materials

Several approaches have been identified to engineer cell surfaces with polyelectrolyte materials; these generally involve conjugating functional groups to specific cell surface ligands using (1) electrostatic interactions between positively-charged polyelectrolytes and negatively-charged cell surfaces; (2) covalent binding of cargos to the amino groups on cell surfaces; and (3) hydrophobic interactions between polyelectrolyte backbones and cell lipid bilayers. Since many cell surfaces are negatively-charged, electrostatic interactions have been exploited to adsorb polycations (e.g., poly-l-lysine (PLL)) onto cell surfaces. By alternating exposure of cells to polyanions and polycations, cells can be deposited with a controlled number, thickness, and concentration of cargo layers. However, direct deposition of polycations onto the cell surfaces can result in cell membrane damage that hinders the translational potential [44].

Polyelectrolyte materials can also be attached to cell surfaces via covalent binding with the free amine-groups on cell membranes. For example, polyelectrolytes containing *N*-Hydroxysuccinimide groups covalently bind to the cell membrane without impairing cell viability [45]. However, many cell membrane proteins also contain amine groups, so non-specific binding that can disrupt normal cellular function is a risk. Lastly, hydrophobic interactions between polyelectrolytes and cell surfaces can be harnessed for cell surface engineering [46]. Most polyelectrolytes contain a hydrophobic backbone, which can interact with the cell membrane’s lipid bilayers. In this technique, there are no direct targeting sites, and the material is applied to the entire cell surfaces, virtually encapsulating the cells. The above techniques established the opportunity to engineer cell surfaces with polyelectrolytes for translational purposes. Table 1 summarizes six major areas where polyelectrolytes are being used to engineer cells for translational medicine, including adding novel functions to viable cells, cell therapy, tissue engineering, cell-based drug delivery, sensing and tracking, and immune modulation.

## 2. Translational Applications of Polyelectrolyte-Based Cell Surface Engineering

### 2.1 Conferring New Functions without Limiting Viability

Polyelectrolyte materials offer a versatile platform to actively modulate cell functions in a synthetic environment. The simplest approach in cell surface engineering is to attach polycations onto negatively charged cell surfaces via electrostatic interactions. Based on this concept, PEMs have been assembled onto cell surfaces to form a synthetic shell for a range of biomedical applications. While the technology is adaptable, direct deposition of polycations, such as poly(allylamine hydrochloride) (PAH), poly(diallyldimethylammonium) (PDAC), and PLL, onto cell surfaces can dramatically decrease cell viability. This can occur in a short period of time (<1 h), damaging cell membranes with pores that allow for the drainage of intracellular components, such as lactose dehydrogenase [44,48].

**Figure 2:**
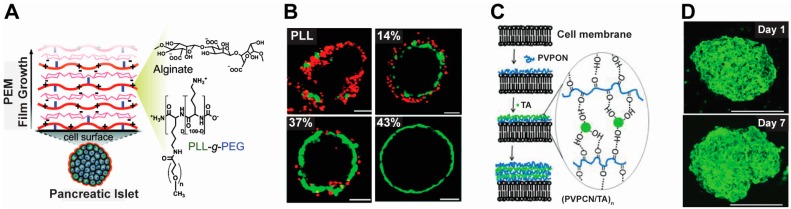
Technologies that are used to minimize the toxicity of polyelectrolyte materials to cells. (**A**) PLL was tailored with PEG units to form PLL-PEG copolymer to reduce toxicity [47]; (**B**) Different levels of PEG units in PLL-PEG copolymer yielded different islet survival rates. Cells were cultured with 80 μm PLL-PEG copolymer and then stained with Calcein AM (green, viable) and ethidium homodimer (red, nonviable) to assess viability. Unmodified PLL resulted in low viability, but islet cell viability increased with a higher level of PEG units. The scale bars are 150 μm. Adapted with permission from [47]; (**C**) PVPON and TA were used in non-toxic PEMs for engineering islet cell surfaces. Adapted with permission from [43]; (**D**) Islet cells modified with (PVPON/TA)_4_ on the day of coating (Day 1) and after 7 days of culture (Day 7). Cells were stained with propidium iodide (PI) and fluorescein to indicate viability. On Day 7, most of the cells were green in color, indicating significant viability. Scale bars are 150 μm. Adapted with permission from [44].

Several strategies have been developed to minimize the damage of polycations on cell viability. For example, Krol et al. used polycations (i.e., PAH) and polyanions (i.e., PSS) to form PEMs on islet surfaces to protect from immune attack, though the coatings still created some toxic impacts [73]. Wilson et al. employed PEM shells composed of polyethylene glycol-poly(l-lysine) (PEG-PLL) and alginate to modify islet cell surfaces for the treatment of diabetes (Figure 2A) [47]. During diabetes, islets—cells that make insulin—can be attacked and destroyed by the immune system. In this study, the authors demonstrated that PEG-PLL/Alginate PEM shells reduced toxicity to islet cells compared to PLL/Alginate PEM shells. In contrast, PEMs composed of PLL and alginate resulted in significant toxicity (Figure 2B). To understand the toxicity of PEG-PLL copolymers on the cells, the authors synthesized a library of PEG-PLL copolymers with varying numbers of PEG units. Viability studies revealed toxicity of PEG-PLL was associated with the length of PEG units within the copolymer and that a longer PEG chain yielded a decrease in toxicity (Figure 2B). The authors hypothesized the PEG chain drove a conformational change during the formation of PEM shells on cell surfaces, allowing the cytocompatible part of the copolymer (i.e., PEG units) to attach to the cell surfaces [47].

**Figure 3:**
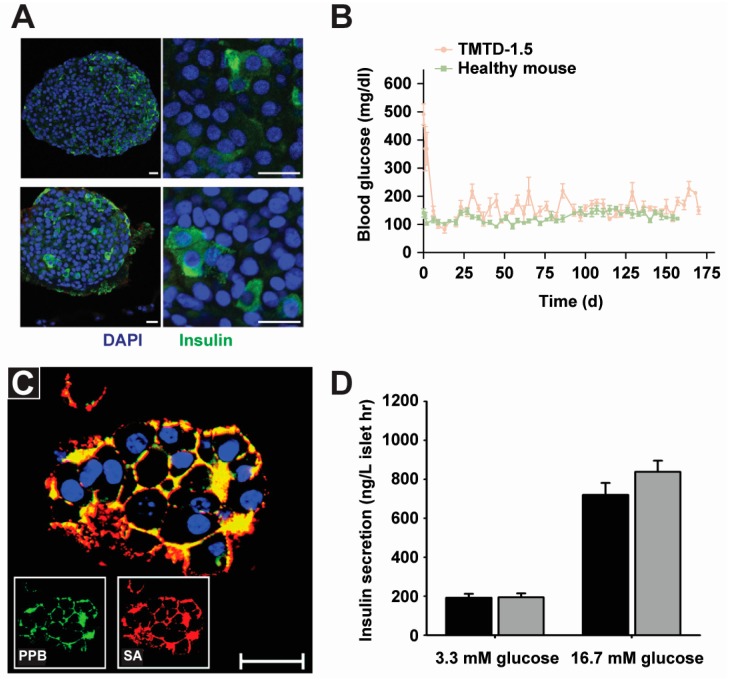
Polyelectrolyte materials are used to engineer the surfaces of islet cells to enhance cell therapy for diabetes. (**A**) Islet cells encapsulated in an alginate matrix before implantation (top) and after retrieval at 90 days post implantation (bottom) from C57BL/6J mice with diabetes induced by a drug (streptozotocin). Cells maintained insulin secretion throughout the experiment. Nuclei are stained blue and insulin is stained green. Scale bars are 20 µm [55]; (**B**) Islet cells encapsulated in an alginate matrix (STA TMTD-1.5) sustained long-term euglycemia in diabetic mice. Adapted with permission from [55]; (**C**) PEM shells composed of PLL-PEG and streptavidin (SA) were used to engineer islet cells for diabetes treatment. Cell nuclei are shown in blue. The green color is PLL-PEG conjugated with FITC, and the red color is Cy3-labeled SA. The yellow color is an overlay of the fluorescently-tagged PLL-PEG and SA. Confocal microscopy imaging revealed PEMs were localized on the cell surface. Scale bar is 50 µm [45]; (**D**) Irrespective of concentration, both uncoated (black bar) and PEM-coated islets (gray bar) responded to glucose stimulation by secreting insulin, indicating PEMs coating did not affect islets functions. Adapted with permission from [45].

In addition to electrostatic interactions, the Tsukruk group explored hydrogen bonding to form PEMs for surface engineering of islets for diabetes treatment [42]. In this work, PEMs were composed of poly(*N*-vinylpyrrolidone) (PVPON) and tannic acid (TA) (Figure 2C). Interestingly, TA has an anti-oxidative property that protect cells against damage from oxidative molecules. The authors demonstrated that modifying islet cells with PVPON/TA PEMs did not affect viability or normal cellular functions such as insulin secretion (Figure 2D, top). In contrast, PEM shells composed of PAH/PSS were toxic (˃87% cell death) and impaired normal cell functions, such as the expression of cytosolic enzymes [44]. In vitro, under both low (3.3 mM) and high (16.7 mM) glucose stimulation, both uncoated and PEM-coated islets responded by secreting a statistically similar level of insulin (Figure 3D) [44]. These studies revealed that PVPON/TA coated cells maintained viability during the 7-day culture period (Figure 2D, bottom). This is desirable for islet cell therapy during diabetic disease [42].

Keeping cells alive during cell surface modification is a prerequisite for most biomedical applications. The above studies demonstrated that polyelectrolyte materials provide a material library for engineering cell surfaces while maintaining viability. Additionally, recent studies reveal polyelectrolyte structure and molecular weight can influence performance for in vivo applications, especially in the immune system. For example, Andorko et al. demonstrated that poly(β-amino esters)—polycationic materials—activate dendritic cells and influence antigen presentation in a molecular weight-dependent fashion in both cells and mice [74]. While this study was in the context of vaccine carrier design, it also highlights the potential for an optimizing polyelectrolyte-materials for cell surface interactions by altering the structure and molecular weight of the modifying polymers.

Incorporating functional materials into PEM shells can alter cell growth and lead to unique capabilities that cannot be achieved with unmodified cells. For example, incorporating silica nanoparticles into PEM shells slows cell growth [49,50]. Yang et al. used PEMs composed of polyelectrolytes and silica nanoparticles to modify yeast cells. This approach significantly reduced yeast cell growth rate while maintaining viability over time [49]. In a subsequent study, the same group demonstrated the PEM shells enhanced cellular defense mechanisms against heat. After infrared lamp exposure for 35 min, 80% of silica-PEM shell coated cells were still alive, while less than 5% of uncoated cells were viable [50]. The incorporation of functional materials into PEM shells over cell surfaces can also create cells with novel functionalities. For example, incorporating carbon nanotubes and graphene flakes into PEM shells generates conductive cells that can be used for cell-based electrical devices and sensors [51,52]. Similarly, by adding magnetic nanostructures into PEM shells, a magnetic field can be used to control the flow and placement of cells for generating magnetically controlled 3D cellular structures [53,54]. These PEM-functionalized cells have unique clinical applications: under a magnetic field, cells with PEM shells containing magnetic materials might be guided to specific organs or tissues in vivo for different therapeutic purposes.

### 2.2. Cell Therapy

Cell therapy is a quickly expanding field in translational medicine [75,76]. Clinical and pre-clinical cell therapies usually rely on injecting or transplanting live cells, either autologous or allogeneic (i.e., same species), into a recipient directly to treat disease. The cells are often pretreated or genetically modified ex vivo (i.e., outside the host) to enhance their performance in vivo. In addition, a supportive therapeutic cargo can be co-administered with the cells to provide a beneficial microenvironment [77,78,79,80]. Beyond these existing approaches, surface engineering of therapeutic cells with synthetic materials, such as polyelectrolytes, has received significant attention in recent years [27,81,82]. In this section, we will discuss polyelectrolyte-based cell surface engineering approaches for cell therapy.

Clinical and preclinical diabetes trials have sought to replace conventional insulin injections with islet implantation. While islet transplantation has shown promising results, a significant barrier is that the infused islets do not maintain long term function. This is due to a combination of delayed revascularization, host inflammatory response, and innate and adaptive immune responses [81,83]. As discussed in the Section 2.1, biomaterials can protect islet cells from these issues by encapsulation, for example, islets can be contained in a bulk hydrogel material for protection and increased survival time. Additional recent studies have also elucidated the therapeutic impacts in animal models. For example, in a study by Vegas et al. islet cells were encapsulated in alginate for type 1 diabetes treatment. This study showed the alginate helped block an immune attack by the recipient animal’s immune systems; the transplanted cells remained alive and functional for 174 days, suggesting long-term glucose control (Figure 3A,B) [55]. Other groups have also used alginate and additional PEM materials in pre-clinical and clinical trials for islet transplants to treat diabetes [84,85].

Encapsulating islet cells in bulk materials can hinder transport through the portal vein of the liver—another challenge in pancreatic islet transplantation [86]. Compared to encapsulating islets in bulk materials, PEM shells can help overcome these transport issues, and also provide some unique advantages for reducing implantation size, improving trafficking efficiency, and increasing nutrient transport. Wilson et al. engineered islets surface with PEG-PLL copolymers (Figure 3C) [44]. In a mouse model, they observed individual islet cells distributed throughout the liver, demonstrating the stability of PEM shells even after entry to the liver [44]. In a drug-induced mouse diabetes model, transplantation of PEM-coated islets conferred a 2-fold reduction from a diabetic state to a euglycemia—a normal level of glucose in the blood, compared to uncoated islets—indicating islet implants reversed diabetes to a normal state in mice. This work is significant because it is the first study to document the survival and function of PEM-coated cells after in vivo transplantation [47].

The idea of implanting islet cells for diabetes treatments has existed for over 30 years. Most techniques have focused on developing a semipermeable passive membrane that can protect the islets from the complicated in vivo environment within the recipient [87]. Polyelectrolyte-based cell surface engineering can contribute to this idea in several ways. First, PEMs are versatile in their properties, including composition, mechanical properties, and permeability; thus, PEMs can be used to optimize the microenvironment for islets. Second, PEM shells can incorporate a defined number of islets for implantation; some studies have demonstrated the feasibility of incorporating single or multiple islets within PEM shells [88]. Other studies also show the number of cells transplanted impact the efficacy of the cell therapy [89]. Together, these results indicate that, in clinical and pre-clinical trials, islet implantation therapy can be improved by optimizing the number of islets encapsulated and implanted. Thus, the unique ability of PEMs to control these parameters is an important area for diabetes research.

Along these same lines of control, the Jewell Lab has developed novel PEMs composed entirely from immune cargos, such as antigenic peptide and toll-like receptor ligands—RNA or DNA that exists in virus or bacteria but not in human—to promote or block specific immune functions [36,39,76,90]. While these materials have not yet been coated on cells, using functional immune PEMs (iPEMs) to modify islet surfaces could provide new opportunities to help control the interactions between the transplanted islets and the recipient’s immune system.

**Figure 4:**
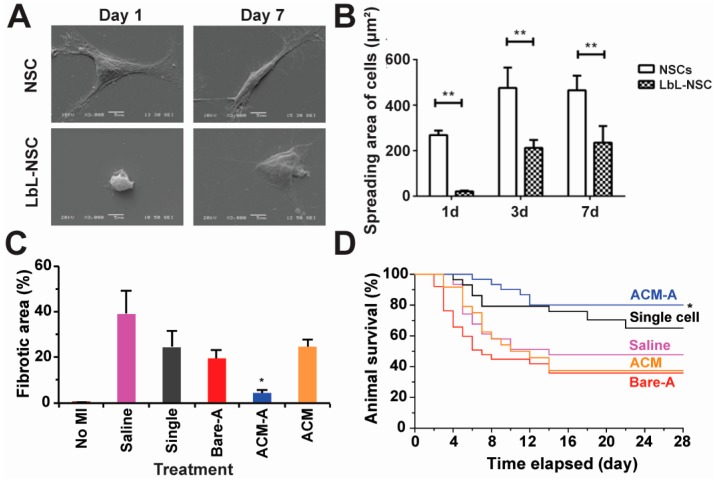
Stem cells are engineered with polyelectrolyte materials on their surfaces for therapeutic purposes. (**A**) Morphological changes of uncoated NSCs (top) and PEMs coated NSCs (bottom) on days 1 and 7. The NSCs were characterized with scanning electron microscopy. Gelatin and alginate were used to form PEMs on NSCs surfaces [56]; (**B**) The spread area of uncoated and PEMs coated NSCs on days 1, 3 and 7. The PEM coated NSCs displayed decreased spread area compared to uncoated cells over time. ** *p* < 0.01. Adapted with permission from [56]; (**C**) The injection of embryonic stem cells encapsulated in alginate-chitosan micromatrix (ACM-A) improved heart function by reducing fibrosis in mice with myocardial infarction. The mice treated with ACM-A showed the least evidence of fibrosis in heart tissue compared to other groups (Saline, Single stem cell (Single), Bare differentiated stem cells (Bare-A) and Matrix only (ACM)) [89]; (**D**) In a survival study, ACM-A treated mice had the longest survival compared to other groups. Adapted with permission from [89]. *: *p* < 0.05; **: *p* < 0.01 in all figures.

Beyond islet cells, surface engineering is being explored to modify stem cells for treating central nervous system disorders [56]. In one study, Li et al. coated individual rat neural stem cells (NSCs) with PEM shells composed of gelatin, alginate and a functional cargo (i.e., insulin-like growth factor-1) without affecting cell viability (Figure 4A) [56]. NSCs are self-renewable, multi-potent cells that produce the neurons and glia in the nervous system during animal embryonic development. The insulin-like growth factor-1 within the PEMs was released in a pH- and time-dependent manner to promote NSC proliferation. While NSCs coated with control PEM lacking growth factors exhibited reduced spreading compared to uncoated NSCs, this morphology difference did not correlate with differences in proliferation. In contrast adding insulin-like growth factor-1 to the PEM shells enhanced NSCs spreading compared to the uncoated NSCs over a 7-day culture period (Figure 4B) [56].

In another recent report, Zhao and coworkers incorporated pluripotent stem cells (PSCs) into a matrix composed of alginate and chitosan for myocardial infarction (i.e., heart attack) treatment. They found that the polyelectrolyte matrix reduced cell damage during injection partially due to the enhanced cell surface modulus from the polyelectrolyte matrix. In mice, cells protected with polyelectrolyte matrix maintained higher survival levels than non-protected cells (50% vs. 20%). Importantly, the polyelectrolyte-modified PSCs significantly reduced the fibrotic area that often thickens as a result of damage during heart attack (Figure 4C). Further, with respect to efficacy, mice receiving polyelectrolyte-protected stem cells showed enhanced survival (Figure 4D) [89]. Similarly, other stem cells such as murine mesenchymal stem cells have been encapsulated in PEMs for surface engineering [91]. These successful examples support the use of PEMs to engineer stem cells for new therapies.

Early clinical studies have made significant progress in using different types of stem cells to address a spectrum of diseases. For example, Limbal stem cells are registered in Europe for the treatment of eye burns [92]. In another recent study in Canada and New Zealand, mesenchymal stem cells are being used as potential treatments for transplant rejection in pediatric patients. Beyond these two examples, a number of other applications are in clinical trials [93]. However, challenges exist in most stem cell-based therapies, including inadequate survival, homing, proliferation, and differentiation of the cells [93,94]. The two examples summarized in Figure 4C,D demonstrate the feasibility of using polyelectrolytes to engineer stem cells while maintaining their functions. Polyelectrolyte-based cell surface engineering can also enable new strategies to address other challenges in stem cell therapy. For example, the versatility of LbL allows stem cell homing factors to be incorporated into the PEM shells coated on stem cells to direct migration. This idea might solve the challenges associated with stem cell implantation on homing to heart, pancreas, or other target tissues. Similarly, other signals, including drug protein therapeutics, can be incorporated into PEM shells to promote stem cell function after implantation.

### 2.3. Tissue Engineering

Many tissue engineering applications involve assembling cells into solid supports or scaffolds that promote adhesion, proliferation, and in some cases, polarization toward desired functions. Hydrogels are one of the most commonly used supports, but surface modification is often necessary for cell adhesion. Despite their wide-spread use, a potential issue with hydrogel-based tissue engineering is the failure to promote the effective diffusion of the necessary chemical and biological cues into the core part of the gel [95]. The inherent and tunable permeability of polyelectrolyte materials offers new strategies to address this limitation of cell surface modification.

**Figure 5:**
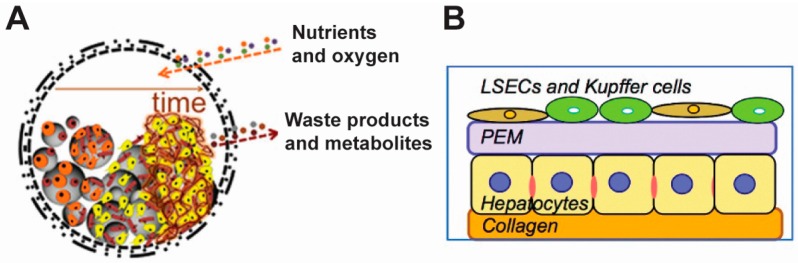
Polyelectrolyte materials are used to engineer cell surfaces to form constructs for tissue engineering purposes. (**A**) A liquefied multilayer hierarchical capsule can encapsulate cells and polymer particles for tissue engineering. The polyelectrolyte capsules were semipermeable, allowing the trafficking of nutrients and waste. The polymer particles were surface modified to promote cell adhesion. Adapted with permission from [10]; (**B**) Cellular constructs with layered structures were designed to mimic real tissues. The first layer of cells (e.g., hepatocytes) was attached to a collagen layer. This was followed by a PEMs layer, onto which another layer of cells (e.g., LESCs and Kupffer cells) was attached. Adapted with permission from [57].

Correia et al. have developed a liquefied multilayer hierarchical capsule that can encapsulate cells and microparticles [10]. The capsules are composed of chitosan and alginate—two biocompatible materials—and are semipermeable to nutrients, oxygen, wastes and metabolites. Microparticles made from poly(l-lactic acid) (PLLA) were surface modified to promote cell attachment (Figure 5A). This design allowed tailoring of capsule properties, such as permeability and mechanical integrity. In addition, different types of cells could be cultured within the semi-closed tissue environment to study the interactions among the cells. During a set of follow-up studies, semi-permeable reservoirs were used to co-culture multi-phenotypic cells to resemble the environment of bones [8]. In this work, capsules were synthesized by LbL assembly using PLL, alginate, and chitosan. Stem cells isolated from adipose tissue (i.e., fat) and endothelial cells from the inside of blood vessels were then adhered to collagen I-modified PLLA particles. This particle/cell matrix was then encapsulated in the PEM capsule. The co-culture environment promoted the formation of bone (i.e., osteogenesis) both with and without osteogenic differentiation factors, as evidenced by the increased activity of matrix mineralization and the up-regulation of genes associated with osteogenesis [8]. Using a similar device, this same group demonstrated that adipose stem cells could undergo self-regulated cartilage development (i.e., chondrogenesis) without the addition of transforming growth factor beta 3 (TGF-β3). TGF-β3 is a growth factor that promotes differentiation of chondrocytes [9]. These results demonstrate the feasibility of using cell surface engineering techniques for chondrogenesis.

Another key goal of tissue engineering is to design cellular constructs with layered structures that mimic real tissues. Rajagopalan et al. developed multilayered cellular constructs by alternatingly depositing hepatocytes (i.e., liver cells) and biocompatible polyelectrolytes (Figure 5B) [57]. Using this technique, the authors designed three-dimensional (3D) structures composed of different architectures: hepatocyte–PEMs–hepatocyte, hepatocyte–PEMs–endothelial cell, and hepatocyte–PEMs–fibroblast constructs. In this system, the PEMs serve a dual purpose: (1) as a surface to culture hepatocytes, to support maintenance of morphology, cytoskeletal structure, and liver-specific functions; and (2) as an adhesive surface for attachment of a subsequent layer of cells to support multi-level cellular structure. This technique is versatile, allowing for the assembly of multiple types of cells into a layered structure that better mimics tissue. More specifically, the authors constructed a layered 3D cellular architecture composed of different types of cells—primary rat hepatocytes and rat liver sinusoidal endothelial cells (rLSECs)) separated by PEMs built from chitosan and hyaluronic acid. In a co-culture experiment, the hepatocytes and rLSECs maintained their key phenotypic characteristics for 12 days. In contrast, rLSECs cultured alone in the 3D construct lost the target phenotypes within 3 days. Interestingly, in a co-culture with both types of cells, the authors observed an increase in the production of albumin and bile canaliculi—a thin tube for collecting bile secreted by liver cells. These molecules are signals used as markers to assess performance of actual liver tissue [58]. In subsequent work, Rajagopalan co-cultured three types of cells (i.e., hepatocytes, rLSECs, and Kupffer cells) in the 3D constructs to build an organotypic liver model [59]. The advantage in this 3D design is that multiple types of cells could be co-cultured in a layered structure, without loss of individual phenotypes—an important requirement for developing artificial tissues. This system also provides a route to study and understand how cell-cell interactions impact the role of toxins in liver. In a broader sense, such strategies could be used to build other tissues that are composed of different types of cells to provide new fundamental knowledge of tissue engineering and scaffold design and performance.

**Figure 6:**
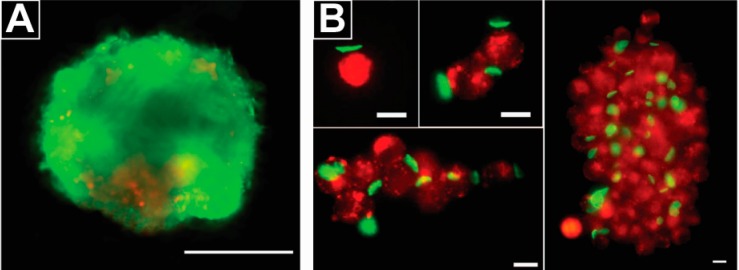
Polyelectrolyte-based cell surface engineering is used in the development of new technology for forming cellular aggregates. (**A**) A thermo-responsive glycopolymer accelerated the formation of cellular aggregates [62]. The cellular aggregates were stained using a live (green)/dead (red) assay to test viability. Scale bar is 200 µm. Adapted with permission from; (**B**) A thin PEM patch (green) was utilized to engineer B cell surfaces in order to form cellular aggregates with a controllable numbers of B cells (red). By varying the cell/patch ratios, cellular aggregates with different number of cells were formed. Scale bars are 10 µm. Adapted with permission from [64].

Cell surface engineering with polymers also presents a powerful approach to promote or control cell aggregates and adhesion for tissue engineering. Pasparakis et al. used a thermo-responsive polyelectrolyte to assemble cells into cellular aggregates/spheroids in a kinetically-controlled manner (Figure 6A) [61,62]. Compared to untreated controls, the use of a glycopolymer accelerated cellular aggregate formation by at least 24 h. In this technique, the authors discovered cell viability was affected by the number of cells used to form the cellular aggregates/spheroids, where aggregates/spheroids produced from a high number of cells had a lower survival rate. The Rubner lab reported a different approach to assemble cells into aggregates using a PEM patch of nanoscale thickness and microscale diameter prepared by photolithography (Figure 6B) [63,64]. This work demonstrated that PEM patches could be used to assemble a controlled number of cells into aggregates, while maintaining cell viability (Figure 6B). Zhang et al. developed a patch composed of PEMs for engineering cell surfaces using soft lithography techniques. These PEM patches served as adhesive components to attached different cell types together, creating a platform for studying cell aggregation and cell-cell interactions [65].

### 2.4. Cell Tracking and Sensing

Cell surface engineering can be used to label cells for the purpose of tracking and sensing both in vitro and in vivo. Zhang et al. functionalized cell surfaces with a disk-shaped PEMs patch composed of polyelectrolyte and gold nanoparticles for surface-enhanced Raman scattering (SERS)-based cell tracking [71]. Gold nanoparticles within the PEM patches were labeled with different Raman reporter molecules for labeling and tracking of multiple types of cells. During Raman spectroscopy, the patch generated SERS signals characteristic of the reporters carried by the gold nanoparticles. This study revealed that varying the number of gold nanoparticle layers in the PEMs could change the intensity of the signal for cell tracking [71]. Fakhrullin et al. functionalized fungal cells with polyelectrolytes and gold or silver nanoparticles for SERS-based cellular analysis. In this work, surface functionalization was used to view the interaction between the nanoparticles and cell walls at different locations [72]. Kahraman et al. modified the surface of Gram-negative and Gram-positive bacteria with PEMs composed of gold nanoparticles and polyelectrolytes to enable SERS-based bacteria identification of different classes of bacteria. This strategy generated new knowledge of bacterial cell chemistry and how bacteria interact with nanoparticles [72]. The above studies demonstrated that cell-tracking materials, such as fluorescent dyes and Raman reporters, can be incorporated into a PEM shell for application to cell surfaces. Using polyelectrolyte-based cell surface engineering, this approach might also allow real-time in vivo monitoring of cell trafficking and homing to particular tissues.

### 2.5. Cell-Based Drug Delivery

In clinical trials, several types of cells are being investigated as drug delivery vehicles, such as erythrocytes (i.e., red blood cells), for example [96,97,98]. Compared to drug delivery with most particles, cell-based drug delivery has unique advantages, including enhanced in vivo circulation, biocompatibility, decreased side effects, and efficient targeting. However, several drawbacks also arise. For example, cargo molecules may diffuse out of the loaded cells, or the loaded molecules within the cells may alter the physiology of the cells being used as carriers [99]. Polyelectrolyte-based cell surface engineering can help address these issues by encapsulating cells with tunable drug-loaded PEM shells or patches.

**Figure 7:**
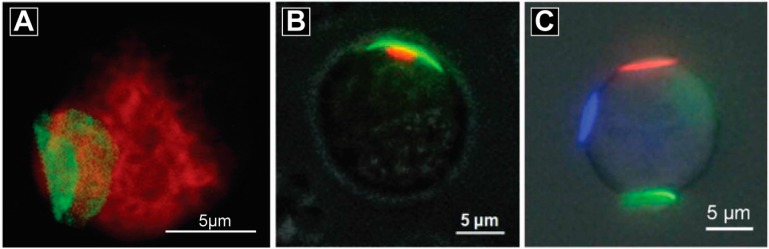
Polyelectrolyte materials are used to engineer cell surfaces for novel cell-based therapy. (**A**) A PEMs patch (green) was attached onto a monocyte (red) surface to treat inflammation. Adapted with permission from [12]; (**B**) A hybrid particle composed of a PEM patch (green) and a thermoplastic dot (red) was attached onto the cell surface for drug delivery purposes. The PEM patches were designed with a negatively charged face and a positively charged face. The positive side was attached onto the cell surface, allowing the unidirectional delivery of therapeutic cargos from the thermoplastic dots. Adapted with permission from [68]; (**C**) Polymer patches loaded with different mock drugs were engineered and attached to cell surfaces for drug delivery purposes. Adapted with permission from [100].

Some of the same PEM cell modification technologies being explored for cell adhesion (see Section 2.3), are also being exploited for drug delivery. For example, the patch developed by the Rubner group can be attached to B cell surfaces—immune cells involved in antibody generation [63]. In early studies, the patch was composed of magnetic nanoparticles and polyelectrolytes, allowing magnetic control of cell trafficking [63]. In a subsequent report, this same platform was used to load and release an anticancer drug, doxorubicin (DOX), from the PEM patches for delivery to cancer cells in culture [67]. An interesting property of these materials is that the coatings, for example, when deposited on phagocytic cells such as macrophages, is that the patch is not internalized [74]. This interesting outcome different compared with conventional spherical particles with the same diameter, which are normally internalized by macrophages [66]. The Rubner and Mitragotri groups also attached PEM patches onto monocyte surfaces to target inflammatory tissues in mice [12]. The PEM-modified monocytes penetrated target tissues (i.e., skins and lungs) and maintained important immune cell functions upon reaching these sites. While no drug was loaded in the PEM patches in this study, this work demonstrated the feasibility of using cell-mediated cell therapies to broadly target inflamed tissues (Figure 7A). Additionally, from a drug delivery perspective, multiple types of drugs can be loaded into patches or other PEM coatings, creating a versatile platform for cell-based drug delivery.

Zhang et al. developed thermoplastic-PEM hybrid particles that were attached onto cell surfaces for unidirectional drug delivery to cells [68]. In this work, a model therapeutic cargo was loaded into the PLGA thermoplastic component and the PEM was used to control cell attachment. In this design, one side of the PEM was negatively charged and the other side was positively charged. The positively charged face of the PEM, attached to the cell surface in a unidirectional manner, allowed controlled delivery of loaded drugs to the cells. The dimensions of the thermoplastic and PEM components, as well as the therapeutic loading level, could be tuned during fabrication to enable well-defined drug delivery (Figure 7B) [68]. Similarly, Xia et al. loaded enzymes into the thermoplastic component that were continuously released from the patch, while maintaining enzymatic activity. By varying the number of patches during cell modification, the number of patches adhered to the cells could be controlled. The authors further demonstrated the feasibility of engineering one cell with several patches each containing a different cargo. This highlights the possibility of delivering multiple drugs to the cells using this approach for cell surface engineering (Figure 7C) [100,101].

While the sections above used a PEM patch on a portion of a cell, fully encapsulating cells with PEMs shells loaded with drugs are also being explored for delivery. Lin et al. employed PEM shells to encapsulate dermal papilla cells—highly specialized mesenchymal cells located in hair follicles—for hair regrowth [69]. The PEM shells were composed of polyelectrolytes and fibroblast growth factor-2—a potent mitosis-promoting factor for fibroblastic cells (i.e., cells providing structural framework in animal tissues). In vitro, the researchers demonstrated PEM shells helped maintain the viability, morphology, and proliferation of the encapsulated cells. In contrast, unmodified dermal papilla cells easily lost their functions when they were cultured in vitro. In mice, PEM-encapsulated dermal papilla cells enhanced hair growth in a hair reconstitution model. Since red-blood cells quickly recycle and pass many biological barriers, they are also being studied as carriers or templates for new drug delivery opportunities. These approaches might also hold potential for cell-mediated drug delivery into other important cells populations, such as leukocytes and stem cells [102].

In the area of cancer, cell-laden microcapsules have also been explored as therapeutics. Sakai et al. for example, engineered cells to express an enzyme that activates a cancer therapeutic [70]. These cells were encapsulated in agarose microparticles and the ability to activate the drug in vitro was confirmed. In mice, the implantation of cell-loaded microcapsules significantly reduced tumor burden compared to cell-free microcapsules. This technique avoided the fast clearance of drug in blood as often encountered during conventional drug delivery.

The above studies illustrate the use of polyelectrolyte materials to engineer cell surfaces for drug delivery purposes. PEM patches or shells can also be used to control drug diffusion from cells compared to cells being used as carriers after loading with a drug. Importantly, entrapping the therapeutic cargos in the PEM patches or shells can help minimize the alteration of cell functions that occurs when drug is loaded directly in cells. Further, PEM structures can incorporate multiple therapeutic cargos and potentially generate synergistic therapeutic effects, with improved targeting through cell homing.

### 2.6. Immune Modulation

The immune system can be activated with stimulatory cues (i.e., antigens or adjuvants) to generate efficient and specific responses against infectious diseases and cancer. In recent years the use of biomaterials for immunotherapies and vaccines has dramatically increased. Many studies demonstrate polymeric materials can activate pro-inflammatory pathways (e.g., inflammasome) as a result of their intrinsic physicochemical properties [74,103,104]. These discoveries motivate the use of polyelectrolyte materials, not only as delivery vehicles, but also as stand-alone immune cargos to polarize immune function. Additionally, the modularity of polyelectrolytes also provides a platform to guide more rational design of vaccines and immunotherapies [105]. Several strategies use bioactive polyelectrolyte materials (i.e., protein, DNA, RNA) to modulate immunity [36,39,76]. In one approach mentioned earlier, foreign/antigenic peptides and nucleic acid molecules that can activate or block immune signaling were assembled into immune iPEMs to treat cancer and autoimmune diseases [36,76]. These iPEMs create a controlled platform for the rational assembly of multiple immune signals, avoiding the complicated intrinsic immunogenicity of many biomaterials. Furthermore, the same platform can be exploited for cell surface engineering. For example, immune cargos could be used to form PEM shells on cell surfaces in order to disarm in vivo immune suppression by organ transplant recipients—a critical issue in this area. Most cell encapsulation designs rely on a passive protective layer to escape rejection of implanted cells. Co-delivery of immunosuppressant drugs with the implanted cells is also commonly used. Despite these strategies, a significant portion of cells are still depleted within the first few days post implantation [7]. Using immune modulatory cargos to form PEM shells on cell surfaces might offer a route to greatly reduce the destruction of implanted cells by a recipient’s immune system.

In addition to emerging technologies such as iPEMs, recent studies have also demonstrated that polyelectrolytes and other materials can assist in avoiding host immune rejection. Both Wilson et al. and Kozlovskaya et al. used PEM shells to protect islet cells from immune rejection during diabetes treatment [42]. In diabetic mice, PEMs coated islet cells escaped immune attack and yielded a transient reversal of diabetes.

One of the most exciting ideas for cancer therapies is a vaccine like-approach in which a patients’ immune system is exposed to fragments of tumor cells to generate T cells that can destroy tumors. For an effective targeting of tumor tissues, tumor associated antigens need to be processed by the immune cells to activate this anti-cancer immunity. However, many cancer antigens are still unidentified and will mutate continuously, making this idea difficult to implement thus far. Toward addressing this hurdle, the De Geest group used PEM-coated melanoma cells (B16-F10) for modulating immunogenicity against cancers. Prior to cellular encapsulation, the cells were exposed to heat to enhance the expression of heat shock proteins—signals that then increased the immunogenicity of the tumor cells, and upon injection, might lead to enhanced anti-tumor immunity [106]. Thus, this work demonstrates that heat shock proteins can be induced and encapsulated within PEM shells as an immune activators for modulating cancer immunity.

Ways to manipulate less specific mechanisms of the immune system are also being developed, for example, the complement system—a series of pathways that help remove bacteria, toxins, and other harmful agents. Leung et al. demonstrated that engineering red blood cells with different concentrations of hyper-branched polyglycerol (HPG) can be used to control which parts of the complement system are activated [3]. In one example introduced above, the Rubner group demonstrated that materials with non-conventional structure (e.g., high aspect ratios) can alter immune cell functions in ways that conventional spherical particles do not [66]. This concept was used to target these populations while maintaining immunomodulatory functions exerted by cells that monocytes differentiate to (e.g., macrophages) [12]. Lastly, PEM shells composed of alginate and PLL have been used to encapsulate T cells from mice to treat graft-versus-host disease (GVHD)—a disease often occurring as a result of alloreactivity of donor T cells once in recipients [107]. Compared to uncoated T cells, coated cells reduced GVHD and decreased liver damage in mice.

Together, these studies highlight some of the opportunities to use polyelectrolytes to modulate immune function. This concept can be expanded to broader applications as well. For example, intra- lymph node injections of myelin self-antigen and a regulatory signal (rapamycin) was recently shown to permanently reverse paralysis associated with Multiple Sclerosis (MS) after just one treatment at the peak of disease [105]. Polyelectrolyte-based cell surface engineering could be used to encapsulate immune cells for deposition in immune tissues for controlling differentiation of lymphocytes toward inflammatory or regulatory populations, depending on the target application.

## 3. Comparison of Polyelectrolytes to Other Cell Surface Engineering Approaches

In addition to polyelectrolyte-based cell surface engineering, other strategies including chemical modification and genetic engineering are also being used to engineer cell surfaces for translational applications [27,108]. Cell surfaces contain functional groups such as amines, sulfhydryl, carboxyl and carbonyl groups that can be chemically modified with biological materials (e.g., proteins, antibodies, lipids, etc.) or synthetic materials (polymers, micro and nano particles, etc.) to control cell interactions [109,110]. For example, the amine groups on cell surfaces can be reacted with *n*-hydroxysuccinimide (NHS) ester. This strategy has been used to couple polyethylene glycol (PEG) onto islet surfaces to prevent the cells from the host immune reactions [111]. Similarly, biotin has been conjugated onto cells modified with streptavidin. Through this strategy, a variety of small molecules and nanoparticles can be conjugated onto cell surfaces. For example, sLe^x^, a selectin glycoprotein ligand, is reacted with stem cell surface proteins that control cell homing through a covalent streptavidin-biotin bridge to improve in vivo targeting of stem cells [112]. Using a similar method, single-stranded DNAs conjugated with fluorescence dyes have been used to modify stem cell surfaces for monitoring interactions between the cells and the tissue environments [1]. Additionally, through chemical functionalization, many drug carrying nanoparticles have been conjugated onto cell surfaces to enhance therapeutic functions. For example, the Irvine group reacted drug-carrying lipid nanoparticles onto cytotoxic T cell surfaces to enhance cells’ killing capacity in mouse cancer models [113]. While these cell surface engineering strategies offer potential for translation, many require more complex chemistry relative to simple electrostatic adsorption, and provide more limited opportunities for specific applications such as altering diffusions of drugs or analytes analytes into and out of cells, cell encapsulation, and engineering biocompatible cell coatings.

Genetic engineering is also used to modulate cell surfaces by introducing or knocking out specific genes or cell receptors [27,111]; this is perhaps the most established technology for changing cell function and surface expression. Many current studies illustrate that genetic engineering of stem cells can improve stem cell survival, migration and recruitment. In one example, Ting and co-workers have introduced biotin groups to specific surface proteins by genetic modification. This capability allows tunable labeling of cell surfaces with streptavidin-conjugated quantum dots [113]. Despite the progress in genetic engineering of cells, there are persistent safety concerns related to the lack of control over the gene locations and the risk of activating oncogenes during gene editing [110]. In another genetic engineering area, newly emerging tools including zinc finger proteins (ZFPs) and clustered regularly interspaced short palindromic repeats (CRISPRs) are allowing repair of diseased genes [114]. The non-viral delivery of mRNA to cytoplasm rather than nucleus also provides a promising way of introducing new genetic information for altering cell surfaces [115]. In these fields, existing challenges include achieving effective gene transfer that do not rely on viral vectors; an advance that could reduce safety risks. Compared to these techniques, polyelectrolyte-based cell surface engineering offer unique advantages and the opportunity to directly modifying cell surfaces with synthetic materials or chemistry for new functionalities.

## 4. Conclusions

The examples above demonstrate the potential polyelectrolyte-based cell surface engineering has for many translational areas. Compared to genetic-based cell surface engineering, polyelectrolyte strategies avoid tedious protocols; instead relying primarily on facile chemical and engineering techniques. Additionally, most polyelectrolyte materials can be used in aqueous solution, thus avoiding the exposure of cells to harsh organic solvents. Polyelectrolytes also offer easy-to-tailor molecular structures, for many material options and flexibility for specific applications. However, realizing these opportunities will require greater emphasis on developing and testing polyelectrolyte cell modification in clinically-meaningful models, and ultimately in human settings. In this article we have highlighted areas where these investments might lead to the biggest gains.

## Figures and Tables

**Table 1:** Opportunities in using polyelectrolyte materials to engineer cell surfaces.

Opportunities	Cell Modification Strategies	References
Viable cells with novel functionalities	Engineering cell surfaces without toxicity	[44,47,48]
	Generating cells with novel functionality	[49,50,51,52,53,54]
Cell therapy	Encapsulating islet cells with bulk materials	[55]
	Encapsulating islet cells with PEM shells	[42]
	Encapsulating stem cells with PEM shells	[56]
Tissue engineering	Generating cell-laden semi-permeable capsules	[8,9,10,11]
	Fabricating liquefied multilayer hierarchical capsules	[57,58,59]
	Assembing cells in an electric filed	[60]
	Forming celluar spheroids with polymers	[61,62]
	Assembling cells with multilayer patches	[63,64,65]
Cell-based drug delivery	Using cells as drug delivery vehiclesEncapsulating cells with drug-loaded PEMsEncapsulating drug secreting cells with capsules	[12,63,66,67][68,69][70]
Sensing and tracking	SERS-based cell sensing and tracking	[71,72]
Immune modulation	Protecting cells from immune responsesModulating complement activation with polymer grafting	[3,42][66]
